# Perceptions and behaviour of pregnant women in socioeconomic deprivation in rural areas. A qualitative study in France

**DOI:** 10.1111/hex.13472

**Published:** 2022-06-15

**Authors:** Aurélie Cabaillot, Marine Lavarenne, Julie Vaure Chiffre, Frédéric Tessieres, Mathilde Vicard Olagne, Catherine Laporte, Philippe Vorilhon

**Affiliations:** ^1^ Département de médecine générale, UFR médecine Université Clermont Auvergne Clermont‐Ferrand France; ^2^ Université Clermont Auvergne UR ACCePPT Clermont‐Ferrand France; ^3^ Université Clermont Auvergne Institut Pascal Clermont‐Ferrand France; ^4^ Direction de la Recherche Cliniique et de l'Innovation CHU Clermont‐Ferrand Clermont‐Ferrand France

**Keywords:** pregnancy, prenatal care, qualitative research, rural areas, semi‐structured interviews, socioeconomic deprivation

## Abstract

**Background:**

Socioeconomic deprivation (SED) is a risk factor for complications during pregnancy and childbirth, the impact of which has been studied poorly in rural areas.

**Aims:**

To explore the perceptions and behaviour of women living in SED in a rural area with regard to their pregnancy follow‐up.

**Methods:**

A qualitative study using semi‐structured individual interviews was carried out in a rural area in central France. To participate, the women had to have an Evaluation of Deprivation and Inequalities in Health Examination Centres deprivation score ≥ 30.17, be living in a rural area and have given birth during the month before the interview. The interviews were analysed using a thematic approach inspired by grounded theory.

**Results:**

Seventeen women were interviewed. The difficulties of life in a rural area were linked to geographical remoteness, travel costs, lack of public services, inadequacy of nearby healthcare and social isolation. In all cases, pregnancy was an additional difficulty. The adaptive capability was related to the presence of an efficient family and social network. Most of the time, any increase in the limitations exceeded the ability to adapt and affected the medical follow‐up of the pregnancy, although follow‐up appointments were rarely abandoned altogether. Perceptions of birth preparation and parenting sessions were often limited to advice on pain management. Due to their affiliation with their rural area or their choice of lifestyle, the women complained only minimally.

**Conclusion:**

Women often minimize any limitations and implement adaptive techniques that make identification by social and medical services more difficult.

**Patient or Public Contribution:**

Eighteen women in SED were contacted by Childhood Medical Protection, midwives and general practitioners practising in rural areas. One woman declined participation and seventeen were interviewed.

## 1 INTRODUCTION

In France, 20% of the population live in rural communities and 5% live in isolated areas.^1^ This is partly the result of an urban exodus that began in the 1980s. In parallel, there has been an increase in the number of young families living in rural areas.^2^ Among the deprived social categories in rural areas are farmers in financial trouble, people who have been victims of deindustrialization, neorurals, young people without qualifications or from broken families and low‐income families searching for cheap accommodation. These categories benefit from minimum social rights.^3^ Social minima are allocated by the government and aim to ensure a minimum income for certain categories of people with low resources.

Rural areas suffer the disadvantage of having fewer medical gynaecologists, obstetrician–gynaecologists, general practitioners (GPs) and midwives.^4^ As was the case in other developed countries, 20% of French maternity units closed between 1998 and 2003, and the hospitals that remain still offer insufficient capacity to handle all pregnancies.^5^ Recommendations made by the French National Health Authority (Haute Autorité de Santé) in 2010 improved pregnancy follow‐up. Nevertheless, access to care for populations characterized by socioeconomic deprivation (SED) remains a matter of grave concern.^6^ The latest national perinatal surveys show that SED has increased recently among pregnant women.^7^

The socioeconomic difficulties of rural compared with urban populations are linked to poor antenatal follow‐up^8^ and more maternal–foetal complications.^9^ The outcomes of pregnancies in women who live in rural areas are characterized by more maternal and perinatal complications.^10^, ^11^

The interplay between life in rural areas and uncertainty of pregnancy follow‐up has been studied only rarely. The objective of this study was to explore the perceptions and behaviour of pregnant women in SED living in rural areas.

## 2 METHODS

### 2.1 Study design

We chose a qualitative method based on an exploratory approach, suitable for collecting information on life experiences. Harsh living conditions and the possible discriminating effect of SED perceived by some women meant that this investigation was conducted via individual interviews. The region chosen for this study was the Auvergne region, consisting of four counties with a population density of half the national average (50 inhabitants/m²),^3^ being more severely affected by SED, at 18.2% in the four counties versus 14.1% for metropolitan France.^12^ The Auvergne region is poor in terms of primary healthcare provision and lacks gynaecologists, midwives and sometimes GPs. The closure of local hospitals, emergency services and nearby maternity hospitals has created additional difficulties of healthcare access for these populations.^13^

A positive Consultative Ethics Opinion was obtained Comité de Protection des Personnes Sud Est VI, Clermont‐Ferrand, IRB 8526.

### 2.2 Participant recruitment

The study was intended to apply to adult women living in rural areas in Auvergne, in SED and having given birth a maximum of 1 month beforehand, as well as being sufficiently fluent in French for the survey. The Evaluation de la Précarité et des Inégalités de santé pour les Centres d'Examen de Santé—Evaluation of Deprivation and Inequalities in Health Examination Centres (EPICES) score was chosen to assess SED because it accounts for the multidimensional nature of SED. EPICES is a validated obstetrics score,^14^ established using an 11‐question questionnaire with binary responses (yes/no) to show a score‐vulnerability‐dependent relationship.^15^ On a scale of 0–100, a score of 30.17 defines the deprivation threshold.

Participants were recruited according to the theoretical sampling technique.^16^ The criteria for the sample included the main indicators of social position, lifestyle and health in connection with the research question, namely age, marital status, socioprofessional category, level of education, financial positioning, geographical isolation (time to the maternity unit ≥30 min), social isolation, parity, planned pregnancy, declaration of pregnancy before 15 weeks of amenorrhoea, high‐risk pregnancy, pregnancy complications and referral to healthcare professionals for pregnancy follow‐up (midwife, GP, medical gynaecologist or obstetrician–gynaecologist). Recruitment took place in all four counties of Auvergne. Childhood Medical Protection (CMP) were contacted, together with midwives and GPs practising in rural areas. An information sheet about the study and methodology used was provided to each woman contacted. An informative questionnaire was completed by each participant before her recruitment, allowing the researchers to calculate the EPICES score and check the variables determining the sociological profile. These sheets were given out by the midwives, GPs and gynaecologists during pregnancy follow‐up or childbirth. Participation in these interviews was voluntary and signed informed consent was obtained. The women were included until data saturation was reached.

The interviews were recorded on a digital voice recorder with the agreement of each interviewee. Face‐to‐face interviews were held in a place of the participants' choosing. The investigator (M. L.) was present as a healthcare student carrying out research on pregnancy follow‐up in rural areas. The interaction between the interviewee and the investigator was established through general questioning on the experiences of pregnancy. The interview guide consisted of open questions covering the main points for discussion. Several probing questions were planned to stimulate the discussion and the association of ideas (Table 1). To promote discussions, the following intervention strategies were used: open questions, follow‐up (nods, interjections and urging sentences), silences, reformulations and contradictions.

**Table 1:** Interview guide

1. *Tell me about your life in the countryside*
− Can you tell me what brought you to live here?
2. *Tell me about your pregnancy*
3. *Has pregnancy been a challenge in your daily life?*
− If that was the case, can you tell me why?
4. *Tell me about your pregnancy medical follow‐up and how you experienced it*
5. *Regarding this follow‐up, which types of health professionals did you meet?*
6. *Tell me about consultations with doctors, midwives, and the maternity ward*
− What interest did they show in you and your baby?
− Have you ever been unable to attend one or more consultations or tests? If so, can you explain why?
− Describe to me the solutions you implemented

### 2.3 Analysis

The interviews were fully transcribed (M. L.) with anonymization of proper nouns by selecting letters in alphabetical order of appearance of the names in the text. Verbal and nonverbal signals were transcribed: speech, behaviour, emotions and intercurrent events. The verbatims were analysed independently (M. L. and J. V. C.).

The analysis was performed without the use of coding software, according to an approach inspired by grounded theory.^17^, ^18^ It was carried out as the data was collected, by constant comparison until saturation. The open analysis benefited from a triangulation by comparing the results of two researchers (M. L. and J. V. C.). It was carried out in three stages:

1. A first longitudinal analysis of each interview was carried out, with the creation of descriptive codes.
2. In a second step, the open codes were grouped into themes.
3. Then by comparison and integration, the themes relevant to the research question were grouped into coherent units, representing different dimensions of the phenomenon studied. In the event of differences in the analysis, a third researcher (A. C.) was consulted. The discussion was enhanced without substantial disagreement.

## 3 RESULTS

Seventeen women were interviewed between January and April 2016; eight interviews took place in the maternity ward and nine at home. Only one woman refused to participate due to her fatigue. Data saturation was reached after 15 interviews. Two additional interviews were performed to confirm the saturation. The EPICES scores varied between 30.18 and 57.39, and the characteristics of the participants are shown in Table 2. Three themes emerged from the analysis.

**Table 2:** Characteristics of the participants

	Score EPICES	Age	Marital status	SPC	Qualification	Financial difficulties	Geographical isolation	Social isolation	P	Planned pregnancy	High‐risk pregnancy	Complicated pregnancy	Healthcare professionals for monitoring the pregnancy
P1	56.81	22	In a relationship	Unemployed	Not specified	Yes	Yes	No	1	Yes	No	No	Freelance MW
P2	39.05	25	In a relationship	Unemployed	NVQ	No	Yes	No	1	No	Yes	Yes	Hospital MW
P3	34.91	32	In a relationship	DLA	Vocational qualification	No	No	Yes	1	No	Yes	Yes	Hospital MW and gynaecologist
P4	39.05	18	Single	Unemployed	Not specified	Yes	Yes	No	1	No	Yes	No	Hospital MW
P5	33.72	19	Single	In training	Vocational qualification	No	No	No	1	Yes	Yes	Yes	Hospital gynaecologist
P6	52.07	28	In a relationship	Unemployed	+2 years' higher education	Yes	Yes	Yes	1	No	Yes	Yes	MW and gynaecologist
P7	34.31	30	In a relationship	Unemployed	Not specified	No	Yes	No	1	Yes	Yes	No	Hospital gynaecologist
P8	31.37	38	In a relationship	On disability	+2 years' higher education	Yes	No	No	2	Yes	Yes	No	Hospital gynaecologist
P9	40.82	32	In a relationship	Home help	NVQ/vocational qualification	No	No	No	4	No	No	No	Freelance gynaecologists
P10	36.69	32	In a relationship	Home help	NVQ	Yes	Yes	No	4	Yes	No	No	Hospital intern and MW
P11	57.39	26	Single	Unemployed	Other	Yes	No	No	1	No	No	No	Hospital MW
P12	40.24	29	In a relationship	Farmer	College	Yes	Yes	No	2	No	No	No	General practitioners
P13	53.85	24	In a relationship	Unemployed	Baccalaureate	Yes	Yes	No	1	Yes	Yes	Yes	Hospital gynaecologist
P14	48.53	28	In a relationship	Unemployed	+2 years' higher education	Yes	Yes	Yes	2	Yes	No	No	Hospital MW
P15	49.12	35	In a relationship	Secretary	Other	Yes	No	Yes	1	Yes	No	No	Interns
P16	53.85	24	In a relationship	Personal care assistant	Baccalaureate	Yes	No	Yes	2	No	Yes	Yes	Hospital gynaecologist
P17	30.18	24	In a relationship	Unemployed	Baccalaureate	Yes	No	No	1	Yes	No	No	Hospital MW

Abbreviation: DLA, disability living allowance; EPICES, Evaluation of Deprivation and Inequalities in Health Examination Centres (Évaluation de la Précarité et des Inégalités de santé pour les Centres d'Examen de Santé); MW, midwife; NVQ, national vocational qualification; P, parity; SPC, socioprofessional category.

### 3.1 Pregnancy: Source of ambivalence

#### 3.1.1 Living in a rural environment: A difficult but assumed choice

For many women, it seems important to appear strong and ‘own’ their choices, despite their rural lives being challenging and requiring endurance. The choice to live in the countryside appeared to mitigate complaints, and difficulties were often trivialized. Some women were reluctant to ask for help even when they needed it. The factors were complex: family roots, convenient housing, professional restrictions, and hope for a better quality of life.

*Our desire to change our life, to be more ecological, to offer that to our children. […] and above all, due to our professional activities*. P12, 29 years old

*I think it's because we were born near here. It's as I said: you need to be born there to stay there and that's that*. P14, 28 years old

For most women, the main advantage of living in a rural area was the low cost of real estate and the healthy environment, which they deemed beneficial to their children. Nevertheless, they also reported the difficulties linked to geographical remoteness, travel costs, lack of public services, shortage of local healthcare professionals and social isolation.

*Obviously when the car breaks down, we postpone (the appointment). We don't have a choice. There's no bus, no train, there's nothing*. P6, 28 years old

#### 3.1.2 Vulnerable but privileged

Pregnancy was presented by most women as a difficult psychological, physical and financial transition. However, for several of them, it was a period of social recognition, offering some respite from the difficulties of everyday life. Unplanned pregnancies occasionally occurred, and in response to them, there was the hope of relieving social isolation, improving social and conjugal recognition or providing the opportunity to improve their financial situation. Their pregnancy was the impetus for some to resume or start gynaecological or even medical examinations that had been lacking. Even though monitoring of the pregnancy was seen as restrictive, it was nevertheless a sign of reassurance.

*I liked being supported and that they paid me compliments in order to put me up on a pedestal. My boyfriend was only thoughtful at the end when it became real*. P2, 25 years old

*Furthermore, you're all alone. Well, now it's not like that anymore: I've met people and with my daughter I'll be able to go for a walk…do things*. P2, 25 years old

*Already it's getting better financially. Before, I was paid by the employment office and the finances weren't very nice. I'm on maternity leave now, so it's true that it's a bit better. I'll stay on maternity leave all the time! it'll be more difficult afterwards*. P13, 24 years old

#### 3.1.3 The experience of pregnancy

For most of the women, pregnancy follow‐up was a satisfactory experience despite its difficulties. Many women hoped to be good mothers to their unborn children. Some expressed negative experiences in a defensive manner demonstrating a distrust of social services, which they regard as a monitoring system that might possibly lead to their children being placed in the care of the social service

*They weren't necessarily happy that I missed all the appointments. As a result, it brought me a lot of trouble: child protection, the regional council, even though it's not my fault!*. P3, 32 years old

### 3.2 Resilience: A coping strategy

#### 3.2.1 Constraints related to the healthcare system

The closures of local maternity wards were criticized by several women. The perinatal centres and the consultation hours of the CMP were not sufficiently accessible for some and were not adequate to overcome the absence of a maternity ward. The opening times were variable and unsuitable, and the healthcare professionals changed from one month to the next. For most of the participants, there were too many healthcare professionals, and they were difficult to identify. There was a feeling that their difficulties were underestimated, and the diversity of opinions of practitioners regarding care was again a source of further anxiety. The consultations seemed short compared to the efforts made by the women to secure appointments. Several of the women were unaware of the free healthcare offered 4 months after pregnancy was confirmed. It was rare that the women were able to follow to the letter the recommendations made by doctors and midwives concerning meals, medical follow‐up or participation in birth preparation and parenting sessions. They made an effort to follow the medical advice, by taking interest in ultrasound examinations and follow‐up, which allowed them to see and hear their baby.

*I explained to him (the obstetrician) that I came from X and that it was a bit far to come back after lunchtime, that I was tired, that I have another child but it wasn't his problem*. P16, 24 years old

*In fact, for the first appointment that I had with him I had to wait for an hour and a half and when I was seen, I wasn't examined. We hardly spoke and for only a few minutes. […] I was very disappointed*. P16, 24 years old

Most of the participants did not complete the birth preparation and parenting sessions, which they felt were optional resources. Those who did complete them shared that they were reassured with regard to the difficulties linked to the distance from their homes to the maternity ward at the end of their pregnancy.

#### 3.2.2 Pregnancy: An additional difficulty

Apart from the course of the pregnancy itself, several women were apprehensive about the consequences and mentioned the fear of losing their job, of being socially stigmatized, of having a complication detrimental to their health and a lack of confidence as far as feeling that they may be a bad mother. They expressed a lack of confidence towards themselves and others. Two women described the emergence of family and conjugal conflicts, which aggravated their feelings of social isolation.

*I was a little distressed to tell my employer because I had just started work […] I did not know how my supervisor was going to react. These days we can't refuse an employment contract*. P16, 24 years old

*The situation was very tense between us. I was quite alone. […] I went back to my mother's because I was separating from my boyfriend. But it wasn't conceivable to stay at my mother's house, and particularly with a child*. P11, 26 years old

#### 3.2.3 Adaptive capacity overtaken by the accumulation of limitations

Frequency of travel, climatic and geographical constraints and distance from maternity wards were sources of anxiety and stress. The absence of public transport, driver licences or access to motor vehicles meant a reliance on the entourage, such as family, friends or neighbours, which was often poorly characterized by some. Single women, in particular, reported their difficulties in adapting to these challenges. The financial costs of transportation were not always explicitly mentioned as a limitation for monitoring but were always present in underlying discussions.

*What scared me was giving birth in the night and that the roads iced over*. P9, 32 years old

*I had lots of appointments in a very short space of time and I couldn't go to all of them because there was no one to take me, no driving licence, lost in the country. […] I always rely on someone*. P3, 32 years old

*We live far away; we have to take our car to reach all these appointments and we can't always pay!*. P17, 24 years old

*The last biological analysis I couldn't do it because I didn't have a car and no family available*. P5, 19 years old

Managing the unexpected left most of the women without solutions because they had already depleted their adaptive capacity.

#### 3.2.4 Make the follow‐up easier

Among the adaptation strategies implemented, all the women stated the need to anticipate the coordination of care and the optimization of travel. They all established a local social network to help where needed during pregnancy follow‐up. The family and social circle were valuable sources of moral and material support, particularly for travel to care facilities. The neorural women, who had no family roots, reported suffering from geographical isolation. Some women highlighted the fact that full reimbursement of medical costs, thanks to medical insurance after the fourth month of pregnancy, helped them meet the additional costs linked to transportation.

*In fact, my parents live near the maternity hospital, at B…., and given that I had no income and that they were a little ill I preferred to stay with them until I ended the pregnancy*. P4, 18 years old

*My mother and sister are at X so within five minutes they are here. If I need them, I call in the rescue*. P11, 26 years old

Many of the women placed some importance on the relational aspect, closeness and availability in their choice of healthcare professionals. For some, websites dedicated to pregnancy allowed them to obtain information and to overcome geographical and social isolation. Some women made use of home visits from midwives; an intervention described as being a major help but not widespread. The pharmacist also helped them by sending their biological samples to the medical testing facility.

*The fact that she (the midwife) came to the house helped me […] that there wasn't the entire journey to do. She offered to do the birthing class here*. P16, 24 years old

*The maternity is far and the doctors are friendly, they try to adapt and I tried to adapt to them as well*. P13, 24 years old

### 3.3 GP as a local solution

Most of the women thought that the follow‐up of their pregnancy was within the competency of the obstetrician and midwife rather than of the GP. Some experienced a reluctance from the GP to follow up on their pregnancy, especially given a tendency to refer them to specialists. The lack of use of ultrasound and foetal monitoring represented an obstacle to pregnancy follow‐up by a GP.

*I didn't ask myself the question because I didn't think that the general practitioners could do pregnancy follow‐up*. P15, 35 years old

*For me, pregnancy is still a gynaecologist […] It's not my doctor […] It's the job of a gynaecologist […] A doctor can always have advice to give us, it's not the same*. P10, 32 years old

Some women placed their trust equally among their GP, midwife and gynaecologist. For most women, it was their GP who made the initial diagnosis of pregnancy, informed them of pregnancy follow‐up and coordinated the follow‐up and directed them, if needed, to the obstetrics team. Several women described the advantages of their GP's involvement, providing a much appreciated local relationship. The GPs gave important psychological support, and their versatility enabled the overall and specific medical follow‐up of the pregnancy by limiting the use of different healthcare professionals. Some women criticized the lack of coordination between other health professionals and their GPs, believing this to be linked to work overload, thereby leading to a negative impact on the quality of follow‐up. Some women found that consultations with their GP were too short and less in‐depth than those with the midwife, and other women did not make appointments at all if the GP happened to be a man.

*There's the interaction, it's somebody (the GP) that I know, in whom I have confidence. […] And then it's, above all, practical, they're nearby*. P15, 35 years old

*She (the GP) knows me, she knows everything about my life. It's true that I can talk a lot with my GP. As soon as something's wrong or that I've a problem, I know that I can count on her*. P9, 32 years old

## 4 DISCUSSION

This study highlighted the difficulties of pregnancy care encountered by women in SED in rural areas. The specific limitations of a rural area were linked to the geographical and social remoteness, travel costs and an inadequate supply of specialist local healthcare. Pregnancy represented an additional limitation related to fears concerning motherhood, loss of employment and the financial difficulties of raising a child. The women regretted the lack of tailoring of follow‐up and the difficulties of adapting to the healthcare system. All the women adapted by mobilizing a local network of family and friends. The social isolation and accumulation of limitations exceeded their adaptive capacity, which had an impact on the medical follow‐up during pregnancy;  they felt that birth preparation and parenting sessions were optional. Their choice of a life in a rural area, more adapted to their needs, appeared to stop them from making any complaints. Pregnancy associated with the difficulties of living in a rural area makes the identification of SED somewhat complicated for healthcare professionals. GPs were still seen as the closest healthcare professionals in both human and geographical terms, although women granted them a limited role during pregnancy follow‐up.^15^ The main strength of the study was the diversity of the participants, which resulted in saturated data. The aim of maximum variability sampling was to illuminate the same issues but from different perspectives. The interviews took place in the maternity ward or at home, which encouraged spontaneity in the conversations. They took place in the month following childbirth to avoid clinical historical bias. The full transcript of the interviews was provided by the investigator who carried out the interviews for reasons of objectivity and reproducibility. The interviews were read and analysed independently by the investigator and an experienced researcher to minimize the number of interpretations and to increase the internal validity of the study.

One of the limitations of the study relates to the mode of recruitment of pregnant women. The recruitment via GPs or midwives may have created a bias in the information provided concerning the representation of pregnancy monitoring and the role of the GP. Another limitation is related to the quality of the male interviewer. Being interviewed by a female researcher may have encouraged more open communication with the participants; however, a female researcher's role as a doctor may have created a bias in the answers. Validation of these results by the participants was not undertaken due to the possible discriminatory nature of the concept of vulnerability.

Few authors have shown interest in the impact of rural life on pregnancy follow‐up, particularly among vulnerable populations. Several studies have analysed rurality and vulnerability, separately. In a Canadian qualitative study in 2006, rural women complained of the financial costs generated by transport, childcare and loss of earnings.^19^ They described their need to adapt to geographical remoteness and winter conditions.

With the closure of numerous rural maternity wards in France between 2002 and 2012, waiting times for access to maternity wards have increased, which has adversely affected medical follow‐up of pregnancies.^10^, ^20^ This impact has particularly been studied in Canada where there has also been, since 2000, a severe decline in the number of maternity wards in rural areas, despite the considered reliability of the healthcare provided.^21^ The isolation linked to rural areas and neighbourhood deprivation are associated with poorer quality of pregnancy follow‐up and an increase in stillbirths, as well as neonatal and postnatal mortality.^22^, ^23^, ^24^ There were several contradictions in the women's statements in the study, however, in that they described real difficulties but did not immediately perceive them as such. There are several possible explanations for this behaviour. Després et al.^25^ found that the use of care was less prevalent among people with SED. They often have a poor opinion of their health, which can accentuate a tendency to deny themselves care.^8^, ^26^ Any pre‐existing material restrictions are accentuated by the medical follow‐up of pregnancy, in which study participants develop adaptive capabilities to best organize their pregnancy follow‐up. The resilience that this represents is an original result of the present study. The study by Brugier et al.^27^ highlighted that the organization of the care pathway is often difficult for vulnerable populations regarding the quality of any prenatal diagnosis.

Our study reveals the importance of the familial and social environment to overcome geographical isolation. Bertin et al.^28^ showed that the deprivation of neighbourhoods in rural areas was associated with a risk of lower weight and head circumference at birth compared with children living in urban areas, irrespective of the socioeconomic conditions of the mothers.

The multiplicity of practitioners involved in pregnancy follow‐up creates confusion through the variety of opinions given, together with the lack of identification of the healthcare professionals involved in the follow‐up. Venditelli et al.^29^ emphasized the importance of helping the women understand the role of each practitioner in pregnancy follow‐up. Women want to be close to their care during pregnancy, and other studies have shown the benefits of home intervention by GPs, midwives and psychologists in rural areas.^30^, ^31^ The participants did not always identify all healthcare professionals, CMP midwives and GPs as possible resources for their follow‐up, implying a need for better coordination between all the healthcare professionals who could be involved with a pregnancy.^32^

Our study highlights the need for investment in strategies for reducing social inequalities in rural areas to improve perinatal health. Women who reside in rural areas should receive high‐quality maternity care as close to home as possible. GPs, who are the closest health professionals, can play a role in identifying these pregnant women in SED. The use of a questionnaire adapted to this identification in this particular context could be helpful.^33^ Responses could alert social services to set up social and behaviour intervention measures. At a second level, it would be interesting to propose the inclusion of women in care networks that include the maternity hospital and the obstetrician, a local perinatal centre, GPs, midwives, private nurses and psychologists. Woman‐ and family‐centred care requires the collaboration and development of clinical skills of different health professionals. In rural areas, GPs are very much involved with the ageing population and chronic diseases. Their potential involvement in pregnancy monitoring is often underestimated by the community and women themselves. Similarly, the investment of midwives is to be favoured, as well as their collaboration with GPs, which is not always self‐evident.^21^, ^34^ Bringing antenatal care closer to these women would allow them to come out of social isolation, provide them with psychological support and avoid the costs and risks of travel to maternity wards.

This requires creating a system that is accessible, affordable, risk‐appropriate, patient‐centred, coordinated, innovative and equitable. In this objective, the data from our study will be used to create a questionnaire to collect the expectations of a large population of women. This will allow us to adapt healthcare networks as accurately as possible to their needs.

## 5 CONCLUSIONS

The difficulties of pregnant women in SED living in rural areas are linked to geographical remoteness, an inadequate supply of specialist local healthcare, fear of additional financial limitations and anxiety about parenthood. In developing numerous adaptive capabilities, they often minimize any limitations and implement adaptive techniques that make identification by social and medical services more difficult. When the adaptive capabilities are exhausted, there is a negative impact on the medical follow‐up of a pregnancy. The women in our study did not approach their GPs due to lack of confidence in his or her abilities, but they appreciated the proximity and psychological support that was offered.

## CONFLICTS OF INTEREST

The authors declare that there are no conflicts of interest.

## AUTHOR CONTRIBUTIONS

Aurélie Cabaillot (first author) contributed to the conception, design, interpretation of data and writing and revising the manuscript. Marine Lavarenne contributed to the conception, design, acquisition of data, analysis, interpretation of data, writing the first manuscript and final approval of the version to be published. Julie V. Chiffre contributed to the conception, design, acquisition of data, analysis and interpretation of data. Frédéric Tessieres contributed to the conception, revising of the manuscript and the final approval of the version to be published. Mathilde V. Olagne contributed to the conception, revising of the manuscript and the final approval of the version to be published. Catherine Laporte contributed to the conception, revising of the manuscript and the final approval of the version to be published. Philippe Vorilhon contributed to the conception, design, writing and revising of the manuscript and final approval of the version to be published.

## Data Availability

The data that support the findings of this study are available from the corresponding author upon reasonable request.
